# Gallbladder Clear Cell Carcinoma: Report of a Rare Case and Literature Review

**DOI:** 10.1155/2023/8104679

**Published:** 2023-06-24

**Authors:** Panayiotis Papatheodorou, Kyriakos Frantzeskou, Kyriaki Konstantinou, Elena Theophanous

**Affiliations:** ^1^General Surgery Department, Nicosia General Hospital, Nicosia, Cyprus; ^2^Histopathology Department, Nicosia General Hospital, Nicosia, Cyprus

## Abstract

Carcinoma of the gallbladder is the most common biliary tract cancer. The majority of gallbladder cancers are adenocarcinomas, whereas clear-cell carcinoma of the gallbladder (CCG) is a rarely recorded variant. Usually, diagnosis is established incidentally after cholecystectomy, performed for another reason. Clinically, the different histological types of carcinomas are impossible to be recognized preoperatively, since they present with a wide and common range of symptoms. We present a male patient who underwent an emergency cholecystectomy due to suspected perforation. After an uneventful postoperative period, the histopathological report led to the diagnosis of CCG, but the surgical margins were infiltrated by the tumor. The patient decided not to proceed with any additional treatment and passed away 8 months after the operation. In conclusion, it is of great necessity to record such unusual cases and enriches global knowledge with information clinically and educationally noteworthy.

## 1. Introduction

Gallbladder cancer (GBC) is the most common cancer of the biliary tract [1]. Its incidence varies among geographical areas and ethnicities. Clear cell carcinoma of the gallbladder is an extremely rare disease, which is categorized by the World Health Organization as a distinct type of gallbladder adenocarcinoma since 1992 [2]. Tyson and Piney reported an irregular tumor found after a cholecystectomy, which is considered to be the first published case [3]. Within the wide spectrum of causative factors of GBC, cholelithiasis is the most prevalent condition also associated with the clear cell subtype [4, 5]. Due to the limited number of cases reported worldwide, no safe conclusions can be derived on whether a different prognosis is expected or an independent treatment approach can be chosen.

## 2. Case Presentation

An 82 years old man presented to the emergency department of our hospital due to acute abdominal pain. He reported that since the previous week, he was experiencing the symptom mentioned above to a mild degree. During the six previous hours, it gradually worsened and shifted to the right upper quadrant. No other associated symptoms were noticed. On clinical examination, the patient's blood pressure was 144/59 mmHg, his pulse rate was 88 beats per minute, his respiration rate was 19 breaths per minute, and his temperature was 36°C. There was rebound tenderness and involuntary guarding on palpation of the right upper quadrant of the abdomen, with Murphy's sign positive all indicative signs of peritonitis. His anamnesis revealed only arterial hypertension, treated with an angiotensin-converting enzyme inhibitor. He had known cholelithiasis for the last 7 years, but because he remained asymptomatic, he never scheduled a surgical evaluation. These initial findings were evidence of acute abdomen; hence the appropriate investigation was initiated.

**Figure 1:**
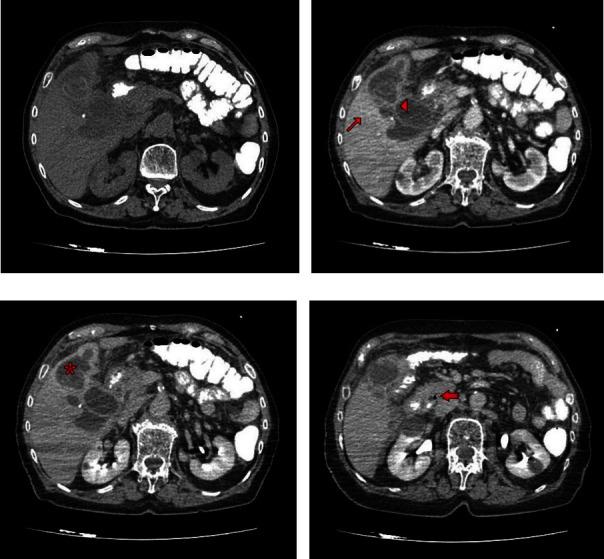
(a) MDCT image with orally administered contrast. (b) Mural thickening (arrowhead), contrast enhancement of the adjacent liver parenchyma (thin arrow). (c) Delayed phase imaging, with extensive pericholecystic and intrahepatic collections (star). (d) Intraluminal air within the common bile duct (arrow).

The results of the patient's blood tests at admission were: white blood cells 9.7 × 10^9^/L (3.91–8.77 × 10^9^/L), hemoglobin 11 g/dL (11.9–15.4 g/dL), platelets 419 × 10^9^×/L (150–450 × 10^9^/L), international normalized ratio 1.05 (0.85–1.15), creatinine 0.87 mg/dL (0.67–1.17 mg/dL), total bilirubin 0.96 mg/dL (0.3–1.20 mg/dL), alkaline phosphatase 182 U/L (30–120 U/L), gamma-glutamyl aminotransferase 137 U/L (9–55 U/L), alanine aminotransferase 27 U/L (3–41 U/L), aspartate aminotransferase 40 U/L (3–38 U/L), amylase 65 U/L (28–100 U/L), and C-reactive protein 428.6 mg/L (0–5 mg/L). The differential diagnosis was broad and included any hepatobiliary pathology, perforated viscus, or intrabdominal infection. Therefore, an abdominal multidetector computed tomography (MDCT) was immediately requested. The imaging results suggested perforation of the gallbladder. The images obtained revealed contrast enhancement of the gallbladder's wall, with irregular thickening, ranging from maximum width of 8 mm at the fundus, to ill-defined foci. Multiple intrahepatic and pericholecystic collections were evident, as well as increased contrast enhancement of the liver parenchyma adjacent to the gallbladder. The common bile duct's diameter was 1.25 cm with intraluminal gas (Figure 1). Even though an abdominal ultrasound is considered to be the first imaging examination of choice for acute cholecystitis and since a diagnosis of an urgent nature was given after performing an MDCT, no further imaging examination was requested.

The severity of the cholecystitis was classified as Grade II [6]. Hence, the patient was transferred for an urgent open cholecystectomy, after the interventional radiology department suggested that any percutaneous drainage would be insufficient, due to the extent of intrabdominal abscesses. A single preoperative dose of piperacillin–tazobactam was given. A gangrenous gallbladder with pericholecystic purulent fluid was the immediate finding after entering the peritoneal cavity. Due to the acute inflammatory process, clear visualization of the peritoneal attachments, the liver bed, and the cystic plate was extremely difficult. The spillage of contents was undesirable, yet unavoidable. However, a retrograde cholecystectomy was safely completed, and both the cystic duct and the artery were ligated, passing each time a 3-0 VICRYL suture. No drainage of the intrahepatic inflammatory collections was performed. The placement of a drain tube in the area was essential. The patient was transferred postoperatively to the surgical ward and at no point was admitted to the intensive care unit. An uneventful recovery was achieved, during which he continued the initial antibiotic regimen. The drain tube was removed on postoperative day (POD) 3 since only a limited quantity of serosanguineous liquid was observed during the previous days. *Escherichia coli* (*E. coli*) was isolated from the bile sample collected during the operation, which was sensitive to the antimicrobial treatment he was receiving. On POD 7, he was discharged, receiving painkillers and low molecular weight heparin for venous thromboembolism prophylaxis. The patient would have been evaluated at the outpatient department, after performing blood tests, to decide if an investigation for possible choledocholithiasis was required.

## 3. Histopathological Findings

**Figure 2:**
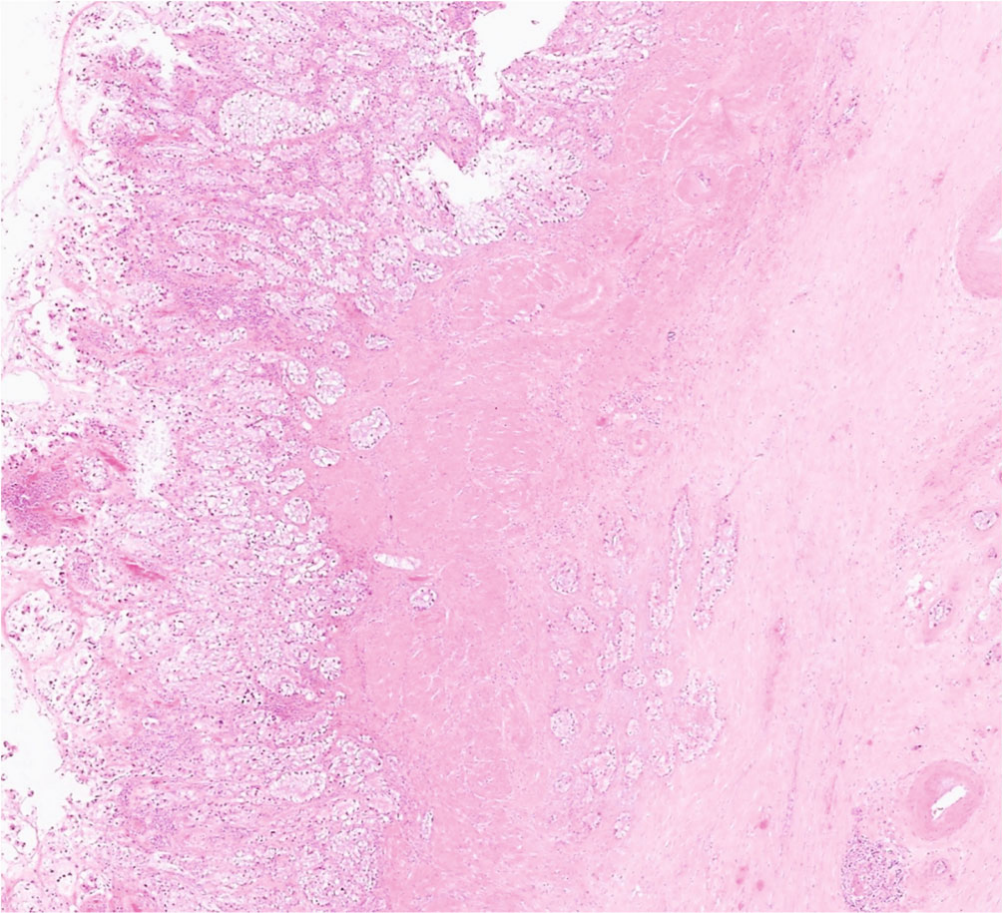
Microscopic examination of H&E stained sections of the tumor, composed of nests, and acini of clear cells, cuboid, and polygonal in shape.

**Figure 3:**
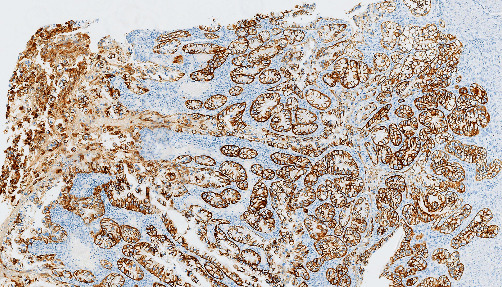
Immunoreactivity of clear cells for CK7.

On gross examination, the gallbladder was opened, measuring 8 cm in length and 3 cm in diameter at the fundus. A small piece of liver parenchyma was attached to the gallbladder. No lymph nodes were retrieved. A diffuse and irregular mural thickening was observed, reaching a maximum width of 1 cm at the fundus. A tan-white colored mucosa was covering the inner surface. The serosa showed necrotic areas with multiple vascular emboli. On microscopic examination of the hematoxylin and eosin (H&E) stained sections, cuboidal and polygonal cells with clear cytoplasm and hyperchromatic nuclei were evident. They consisted of more than 50% of all the tumor cells in the sections examined. The clear cells were arranged in nests and acini. Areas of conventional adenocarcinoma, as well as areas of pyloric and intestinal metaplasia and dysplasia, were also identified (Figure 2). The tumor infiltrated the entire wall of the gallbladder and the cystic duct. It invaded the visceral peritoneum on the peritoneal side and extended into the liver parenchyma attached to the hepatic side. The margins of resection were microscopically positive (R1 resection). The tumor cells reacted positively with cytokeratin 7 (CK7), carcinoembryonic antigen (CEA), cytokeratin 19 (CK19), and the epithelial membrane antigen (EMA; Figure 3). The reaction with cytokeratin 20) was mostly negative. The specimen did not react with cytokeratin 5/6. The carcinoma was classified as T3Nx, according to the eighth edition of the American Joint Committee on Cancer staging manual [7].

## 4. Patient Follow-Up and Outcome

**Figure 4:**
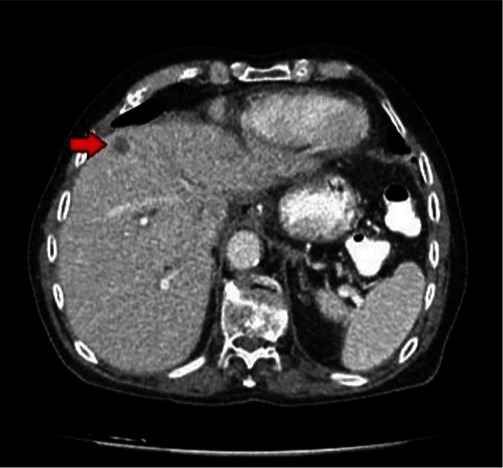
Metastatic lesion (arrow) on liver segment IVa.

On receiving the histopathological report, the patient underwent a thoracic and abdominal MDCT 6 weeks after the operation. A metastatic lesion on liver segment IVa was detected, which was absent on the admission's CT (Figure 4). No visible secondary pulmonary lesions were found. The case was thoroughly discussed with the multidisciplinary team of our hospital. The final decision was to offer chemotherapy. However, the patient and his relatives denied any further treatment because of his advanced age. The patient passed away 8 months after the operation.

## 5. Discussion

Of all subtypes of biliary tract cancer, gallbladder malignancy is the most common [1, 8]. Its incidence is highly variable globally. The highest percentage of this diagnosis is recorded among Native Americans in Chile [9, 10]. High incidence is also reported among the population of India and eastern Asia [11, 12]. In Europe, most cases are documented in Poland and the Czech Republic [9]. GBC affects more frequently occur in female patients compared with male ones at a wide spectrum of ratio values [12, 13]. GBC incidence rates are higher in older patients, especially those over 65 years old [12]. Finally, GBC has a poor prognosis if not diagnosed at an early stage, worse even than other types of cholangiocarcinoma [14]. The short survival of the current case and the brief time between the initial diagnosis and the appearance of the metastatic lesion is an indication of the aggressiveness of the clear-cell carcinoma of the gallbladder (CCG).

Adenocarcinoma is the most common histological type of GBC [15], whereas the clear cell subtype is diagnosed exceedingly rare. Since 1926, which is considered the year that the first case of the clear cell subtype was reported, up to 2021 only 19 cases are published [5, 16–25]. Thus, CCG accounts for less than 1% of all gallbladder malignancies [16]. The largest case series of CCGs, published by Vardaman and Albores-Saavedra suggested that primary CCG predominantly affects women with an average age of 60 years at diagnosis [25]. In comparison with all the previously published cases, the current case is the only one concerning a patient over 80 years old who underwent an emergency cholecystectomy due to possible perforation.

Cholelithiasis is strongly associated with gallbladder carcinoma. Randi et al. calculated a relative risk of 4.9, derived from published cohort studies [13], whereas Graham estimated that gallstones are present in more than half the patients diagnosed with GBC [26]. A controversial aspect of the matter is whether the duration of the presence of cholelithiasis affects the risk of cancer. The longer the irritation of the mucosa, the higher the probability of alteration of the normal cell multiplication is [11, 27]. Contrariwise, Hsing et al. failed to reproduce similar results [28]. In addition, the size of the gallstones is examined whether it contributes to the malignant transformation of the epithelium. Some studies support that the relative risk is proportional to the size of the gallstones [29, 30], which is also the conclusion of a recent systematic review [31]. However, this is not a commonly accepted result [32]. The relatively high heterogeneity of the data published on risk factors for GBC is due to the rarity of the disease and the different genetic profiles of different study groups [31, 32]. Asymptomatic cholelithiasis is not regularly an indication for cholecystectomy, since prophylactic operation is probably beneficial only for patients at high risk of developing GBC. Such risk factors include family history, porcelain gallbladder, gallbladder polyps, anomalous junction of the pancreato-biliary duct, and chronic infection by *Salmonella* sp., *Helicobacter pylori*, or *Clonorchis* sp. [10, 13, 16]. Malignant transformation of ectopic pancreatic tissue of the gallbladder is a very rarely identified condition but is a potential origin of GBC [33]. Among these known causative factors of conventional gallbladder adenocarcinoma, cholelithiasis is the most frequently observed one among the published cases of CCG. To establish a specific etiology of the clear cell subtype adenocarcinoma, a greater number of cases must be accumulated.

Regarding the present case, the patient was incidentally diagnosed with cholelithiasis 7 years before this acute complication. It was not possible to determine whether the initial abdominal ultrasound mentioned any gallbladder polyp and it was not the examination of choice to reveal any anatomic variation of the pancreato-biliary duct junction. Finally, the patient could not recall any relevant family history.

Metaplasia of the epithelium lining of the gallbladder is probably the phenomenon that initiates the development of malignancy [4, 34]. This histological finding is often associated with chronic cholecystitis and is found to carry mutations of the gene p53 [35], which appears early in this process [35, 36]. Since the over-expression of the p53 gene is associated with the manifestation of tumor cells [37], persistent inflammation of the gallbladder can be considered a precursor condition. Somatic mutations of known oncogenes (KRAS, EGFR, and NRAS) were found in GBC cases, but more data are required to establish an association [10].

The most common histological characteristics of CCGs were initially described in the case series of Bittinger et al. [20] and Vardaman and Albores-Saavedra [25]. First, CCG is considered to be the result of the accumulation of compounds, such as glycogen and mucin, in the cytoplasm. This is the reason that these types of cells are not H&E stained. It was also suggested that only the cases in which more than 50% of the total volume of tumor cells show a clear cell differentiation, can be considered as CCGs. The clear cells have distinct borders with little nuclear atypia and normal and abnormal mitoses. In addition, they are arranged in sheets, nests, trabeculae, glands, and small papillary structures and grow in an infiltrative pattern. Finally, many of the cases contain areas of conventional adenocarcinoma [20, 25].

These histological characteristics of CCG are similar to lesions from various other sites, such as renal, endometrial, ovarian, liver, and lung clear cell carcinomas [5, 20]. However, gallbladder neoplasms are rarely proven to be metastatic. The frequency of secondary deposits of renal cell carcinoma to the gallbladder is reported to be 0.6% up to 6.8% in autopsy reviews [38, 39]. This phenomenon was observed by Vardaman and Albores-Saavedra and they suggested that such neoplasms should be considered primary until proven otherwise [25]. During the following years, the immunoreactivity of the CCG was instrumental in establishing an accurate diagnosis. Bittinger et al. initially determined that if CK7 and CEA reactivity is detectable, then the lesion is either primary of the gallbladder or metastatic of a lung mass, excluding clear cell carcinomas of other tissues [20]. In later years, Zhang et al. and Dixit et al. observed that CEA and CK7 were strongly stained in the gallbladder but were negative in nearly all other clear cell carcinomas, including the renal one [5, 17]. More recently, Sarangi et al. added that CCG is also positive for CK19, which is not seen in renal clear cell carcinoma [19]. The differential diagnosis also includes hepatocellular carcinoma (HCC) and metastatic adrenal cortical carcinoma (mACC). However, HCC is rarely or never positive for CK7 [40], and mACC is negative for EMA and CEA and usually negative for cytokeratins [41]. To summarize, combining the immunochemistry results of the present case with the fact that the CT scan performed on admission showed no renal or hepatic lesion, the diagnosis of primary CCG was given.

In conclusion, CCG is an exceptionally rare type of infrequent condition of GBC. The accumulation of knowledge concerning this malignancy can thus primarily be obtained from case reports. An accurate pathological analysis of the specimen is primordial for the identification of different histologic types of GBC, such as CCG. The proper investigation and the final interpretation of all the laboratory and imaging results, can prevent misdiagnosis and offer the patient the appropriate treatment and the analogous prognosis, which in the case of GBC remains unfortunately very poor.

## Data Availability

Data supporting this research article are available from the corresponding author or first author upon reasonable request.
